# Multisensory Audiovisual Processing in Children With a Sensory Processing Disorder (I): Behavioral and Electrophysiological Indices Under Speeded Response Conditions

**DOI:** 10.3389/fnint.2020.00004

**Published:** 2020-02-11

**Authors:** Sophie Molholm, Jeremy W. Murphy, Juliana Bates, Elizabeth M. Ridgway, John J. Foxe

**Affiliations:** ^1^The Cognitive Neurophysiology Laboratory, Department of Pediatrics, Montefiore Medical Center, Albert Einstein College of Medicine, Bronx, NY, United States; ^2^Dominick P. Purpura Department of Neuroscience, Albert Einstein College of Medicin, Bronx, NY, United States; ^3^The Cognitive Neurophysiology Laboratory, The Ernest J. Del Monte Institute for Neuroscience, Department of Neuroscience, University of Rochester School of Medicine and Dentistry, Rochester, NY, United States

**Keywords:** autism spectrum disorders, EEG, multisensory integration, ASD, event-related potential, sensory integration, cross-modal

## Abstract

**Background:**

Maladaptive reactivity to sensory inputs is commonly observed in neurodevelopmental disorders (e.g., autism, ADHD). Little is known, however, about the underlying neural mechanisms. For some children, atypical sensory reactivity is the primary complaint, despite absence of another identifiable neurodevelopmental diagnosis. Studying Sensory Processing Disorder (SPD) may well provide a window into the neuropathology of these symptoms. It has been proposed that a deficit in sensory integration underlies the SPD phenotype, but objective quantification of sensory integration is lacking. Here we used neural and behavioral measures of multisensory integration (MSI), which would be affected by impaired sensory integration and for which there are well accepted objective measures, to test whether failure to integrate across the senses is associated with atypical sensory reactivity in SPD. An autism group served to determine if observed differences were unique to SPD.

**Methods:**

We tested whether children aged 6–16 years with SPD (*N* = 14) integrate multisensory inputs differently from age-matched typically developing controls (TD: *N* = 54), or from children with an autism spectrum disorder (ASD: *N* = 44). Participants performed a simple reaction-time task to the occurrence of auditory, visual, and audiovisual stimuli presented in random order, while high-density recordings of electrical brain activity were made.

**Results:**

Children with SPD showed large reductions in the extent to which they benefited from multisensory inputs compared to TDs. The ASD group showed similarly reduced response speeding to multisensory relative to unisensory inputs. Neural evidence for MSI was seen across all three groups, with the multisensory response differing from the sum of the unisensory responses. *Post hoc* tests suggested the possibility of enhanced MSI in SPD in timeframes consistent with cortical sensory registration (∼60 ms), followed by reduced MSI during a timeframe consistent with object formation (∼130 ms). The ASD group also showed reduced MSI in the later timeframe.

**Conclusion:**

Children with SPD showed reduction in their ability to benefit from redundant audio-visual inputs, similar to children with ASD. Neurophysiological recordings, on the other hand, showed that major indices of MSI were largely intact, although *post hoc* testing pointed to periods of potential differential processing. While these exploratory electrophysiological observations point to potential sensory-perceptual differences in multisensory processing in SPD, it remains equally plausible at this stage that later attentional processing differences may yet prove responsible for the multisensory behavioral deficits uncovered here.

## Introduction

Sensory Processing Disorder (SPD) is characterized by aberrant behavioral responses to sensory inputs (hypo- or hyper- responsiveness) that cause significant disruption to everyday functioning.

Sensory processing disorder may reflect a failure of the nervous system to appropriately modulate and integrate sensory-motor information (Ayres, 1979; Schaaf et al., 2009), with implications for the ability to integrate multisensory inputs. Multisensory inputs from the same object provide redundant and/or complementary cues to its presence, location and identity (Molholm et al., 2004; Fiebelkorn et al., 2011, 2013; Mercier et al., 2015). Clearly then the ability to put such multisensory inputs together lawfully is key to operating optimally within the sensory environment. Conversely, impaired integration across the sensory systems might well lead to a sensory environment that is experienced as overwhelming and/or unmanageable (Foxe and Molholm, 2009; Brandwein et al., 2015), much as seems to be the case with SPD. While SPD has long been associated with atypical sensory processing and integration, and is commonly treated by occupational therapists using *sensory integration therapy* (Miller et al., 2007), there is a shortfall of studies testing the neurobiological underpinnings of dysregulated sensory processing and integration in this population. Nevertheless, the extant literature on SPD is instructive. In one study, diffusion tensor imaging (DTI), which provides an index of the integrity of anatomical connectivity in the brain, was measured in a group of 8–11 year olds (*N* = 16) determined to have SPD based on clinical referral and responses on the Sensory Profile questionnaire (Dunn, 1999). This revealed microstructural white matter differences, in comparison to a neurotypical age-matched control group, that were primarily focused in posterior tracts including left posterior thalamic radiations, and posterior aspects of the corpus callosum, the superior longitudinal fasciculus, and the corona radiata (Owen et al., 2013). Although one must be cautious interpreting the functional significance of these findings, the data are consistent with pathways involved in the intra- and inter- hemispheric processing of sensory information and multisensory integration (MSI). Interestingly however, when the same group looked at magnetoencephalographic recordings of early somatosensory and auditory evoked responses in SPD, they found these to be highly similar to those from a typically developing control group (Demopoulos et al., 2017). In a follow-up study comparing the implicated tracts in SPD versus individuals with autism spectrum disorder (ASD), there was a high degree of similarity between the clinical groups in terms of the posterior tracts, whereas the ASD group was selectively impaired in additionally tested tracts associated with social-emotional processing (Chang et al., 2014). This suggests overlap in the neurobiology of SPD and autism that may relate to atypical responses to the sensory environment. A series of studies from Davies and colleagues, also using clinical referral and a parent based questionnaire (the Sensory Profile) to classify SPD participants, probed the integrity of sensory processing in SPD using non-invasive electrophysiological recordings of brain activity in response to simple auditory stimuli. The resulting data suggested minor differences in sensory processing and sensory adaptation, and in later activity associated with attention at about 300 ms in one study, but not in another (Davies et al., 2009, 2010; Gavin et al., 2011).

The modest amount of data available thus far in SPD, however, do not speak yet to the functional integrity of MSI. Here we used objective and well-characterized behavioral and electrophysiological measures of sensory processing and MSI (Molholm et al., 2002; Brandwein et al., 2013) to assess the integrity of these processes in a sample of individuals with SPD who were diagnosed using both observational and parent report approaches. We focused on individuals with normal-range IQ who exhibited hyper-responsivity to sensory challenges in the tactile, auditory, and/or visual domains. While major sensory processing issues can occur in the absence of another diagnosis, they are also commonly reported in a number of developmental disorders including ASD (Ben-Sasson et al., 2009; Foss-Feig et al., 2012; Schaaf et al., 2013; Tavassoli et al., 2017) and attention deficit/hyperactivity disorder (ADHD) (Reynolds and Lane, 2009). We therefore included a sample of age- and IQ- matched children with a diagnosis of ASD in addition to a typically developing age- and IQ- matched control sample. This allowed us to address whether any identified processing differences were unique to SPD, or if they might instead represent domain-specific deficits that span across clinical diagnoses as previously suggested (Chang et al., 2014). Our working hypothesis was that for individuals with SPD, sensory processing and MSI would be shown to differ from healthy controls during early stages of information processing (<250 ms post stimulus onset), and that information processing differences would be distinct from an ASD group, where the participants were not selected specifically for having sensory hyper-reactivity.

## Materials and Methods

### Participants

Data from 54 individuals with typical development (TD; 22 females) between 6 and 18 years of age (*M* = 9.3; SD = 2.7), 14 individuals with SPD (two females) between the ages of 6 and 16 years of age (*M* = 9.0; SD = 2.9), and 45 individuals with ASD (four females), between the ages of 7 and 16 years of age (*M* = 9.4; SD = 2.0) were analyzed for this study. TD and ASD data were drawn from previously reported datasets (Brandwein et al., 2011, 2013). Groups were matched on performance IQ (PIQ) and age (see Table 1). An analysis of variance (ANOVA) comparing Age and PIQ among the three groups yielded no significant differences among the groups (Age: *F*(2,110) = 0.104, *p* = 0.901; PIQ: *F*(2,110) = 1.391, *p* = 0.253).

**TABLE 1:** Means and standard deviations (in parentheses) for participant data, by diagnostic group.

	TD	ASD	SPD
Age	9.3 (2.7)	9.4 (2.0)	9.0 (2.9)
VIQ	112.5 (11.4)	97.7 (18.9)	104.3 (10.3)
PIQ	105.7 (12.7)	106.8 (18.4)	98.9 (16.7)
FSIQ	110.8 (12.2)	102.3 (18.2)	102.6 (13.8)
N	54	45	14
No. of Males	32	41	12

All children were administered the Wechsler Abbreviated Scales of Intelligence (WASI or WASI-2) to estimate PIQ; Verbal IQ (VIQ); and Full-Scale IQ (FSIQ) are also reported in Table 1. All participants had normal or corrected-to-normal vision and passed a hearing screen. All children were screened for ADHD with the Conners’ Continuous Performance Test (CPT-II).

To determine inclusion in the SPD group, scores from both the Sensory Processing Scale (SPS) Assessment Version 2.0 and The Short Sensory Profile (SSP) were used. Participants were referred to the study by occupational therapists. An occupational therapist (ER) administered the SPS to develop Global Clinical Impressions (GCI) based on direct observation of structured behavior. These were used to determine whether each participant demonstrated “Sensory Overresponsivity” (SOR) in at least one of the visual, tactile, or auditory domains^1^. The SSP questionnaire served to quantify caregivers’ observations of various signs of atypical sensory processing across seven sensory domains. Only three domains were used for inclusion in this study: visual/auditory sensitivity, auditory filtering, and tactile sensitivity. Children included in the SPD group scored in the “Definite Difference” range, indicating a score at least two standard deviations from normed means, in at least one of these three domains and in the overall category that draws on all seven domains. See Table 2 for a breakdown of SSP scores, for all groups (for the 14, 39, and 32 of the participants from the SPD, ASD, and TD groups who completed the testing). ASD served as an exclusionary criterion for the SPD group. SPD participants were screened for autism by a highly trained and ADOS/ADI-R research reliable clinician using clinical judgment; ADOS and/or ADI-R was administered if there was any uncertainty. Inclusion in the ASD group was based on clinical judgment of a psychologist with expertise in the diagnosis of autism, and meeting criteria for an autism spectrum condition on both ADOS-2 and ADI-R assessments performed by a research reliable administrator. Children with ASD and SPD were not excluded for presenting with symptoms of inattention and hyperactivity (based on CPT-II and the DSM-IV ADHD behavioral checklist), since such symptoms are very common in ASD. TD participants were at the appropriate grade for their age, did not present with a history of ASD, ADHD, or other neurological, learning, or neuropsychiatric disorders, were negative on ADHD screens, and did not have a biological first-degree relative with a known developmental disorder. Before participation, informed written consent was obtained from each child’s parent, and verbal or written assent was obtained from each child. The Institutional Review Board of the Albert Einstein College of Medicine approved all procedures. Participants were given $12.00 an hour for their time in the laboratory. All procedures conformed to the ethical standards of the Declaration of Helsinki.

**TABLE 2:** SSP percent classification by group.

Domain	Classification	SPD (%)	ASD (%)	TD (%)
Auditory/Visual Sensitivity	Typical Performance	14.2	38.4	96.8
	Probable Difference	28.5	41	3.2
	Definite Difference	57	20.5	0
Auditory Filtering	Typical Performance	0	15.3	90.6
	Probable Difference	21.4	7.6	9
	Definite Difference	78.5	76.9	0
Tactile	Typical Performance	14.2	40.5	96.8
	Probable Difference	35.7	10.8	3
	Definite Difference	50	48.6	0
Total	Typical Performance	0	16.2	96.6
	Probable Difference	7	16.2	3
	Definite Difference	92.8	67.5	0

### Procedures

Participants sat in a dimly lit, sound attenuated and electrically shielded room. They placed their chin on a chin-rest and maintained central fixation by focusing their eyes on a centrally placed cross, and performed a simple reaction time task in which they responded to presentation of auditory-alone, visual-alone, and audiovisual stimuli with a speeded button press while high-density electroencephalography (EEG) recordings were made. In the auditory-alone condition, a 1000-Hz tone (duration, 60 ms; 75 dB SPL; rise/fall time, 5 ms) was presented from a single Hartman Multimedia JBL Duet speaker located centrally behind the computer monitor from which the visual stimulus was presented. In the visual-alone condition a red disc with a diameter of 3.2 cm (subtending 1.5° in diameter at a viewing distance of 122 cm) appearing on a black background and presented for 60 ms on a monitor (Dell Ultrasharp 1704FTP). During the audiovisual condition, the auditory and visual stimuli were presented simultaneously. In all conditions, participants were instructed to press a button on a response pad (Logitech Wingman Precision) with their right thumb as quickly as possible when they saw the red circle, heard the tone, or saw the circle and heard the tone. The same response key was used for all three stimulus types. Stimulus conditions were presented in random order in blocks of 100 trials, and were presented equiprobably. The interstimulus interval ranged equiprobably and pseudorandomly from 1000 to 3000 ms. Participants completed between 6 and 10 blocks, with the majority completing 10 blocks. To reduce restlessness or fatigue, breaks were encouraged between blocks to help maintain concentration.

### Data Acquisition and Statistical Analysis

#### Behavioral Analyses

Button press responses to the three stimulus conditions acquired during the recording of the EEG were processed offline using Matlab. Mean reaction times (RTs) and standard deviations were calculated for each condition for each participant using a two-step procedure for detecting outlier RT values. First, a hard threshold was applied in which all RTs faster than 150 ms or slower the minimum of the variable ISI (1000 ms) were excluded (to exclude anticipatory responses). Next, since significant inter-subject variability in RT was expected due to a relatively large age-range and inclusion of typically developing and clinical groups, additional thresholds were applied based on each participant’s RT distribution. Specifically, only trials with RTs falling within the inner 95% of an individual’s RT distribution were included. That is, the fastest 2.5% and the slowest 2.5% of RTs within an individual’s distribution were discarded. Using a 95% cutoff to define the time window for acceptable trials allowed us to more accurately capture the range of RTs for each participant, an important factor in calculating the race model (described below). Hits were defined as those trials on which a button press occurred within the individual’s specific 95% RT range. Responses outside of this window were considered misses. Separate 3 × 3 mixed design ANOVAs with factors of Diagnostic Group and Stimulus Condition were performed to assess group differences in RT and hit rate. Planned comparisons between each of the unisensory conditions and the multisensory condition tested for the presence of the “redundant signal effect” [redundant signals effect (RSE): a faster reaction to multisensory than to unisensory stimuli] in the RT data.

##### Testing the race model

Behavioral facilitation for the multisensory condition compared to each of the unisensory conditions may occur simply due to probability summation; therefore, Miller’s race model (Miller, 1982) was implemented. The race model assumes that mean RTs decrease because there are now two inputs (e.g., auditory and visual) to trigger a response, and the fastest “wins the race.” Thus facilitation can be explained in the absence of interaction between the two inputs due to probability summation. However, when there is violation of the race model, it is generally assumed that the unisensory inputs interacted during processing to facilitate RT performance. Miller’s race model (Miller, 1982) places an upper limit on the cumulative probability (CP) of a response at a given latency for redundant signals (i.e., the multisensory condition). For any latency, t, the race model holds when this CP value is less than or equal to the sum of the CP from each of the single target stimulus conditions (the unisensory stimuli). For each individual, the range of valid RTs was calculated across the three stimulus types (auditory-alone, visual-alone, and audiovisual) and divided into quantiles from the 5th to 100th percentile in 5% increments (5, 10,…, 95, 100%). Violations were expected to occur at quantiles representing the shorter RTs because this is when it was most likely that interactions of the visual and auditory inputs would result in the fulfillment of a response criterion before either source alone satisfied the same criterion (Miller, 1982; Ulrich et al., 2007). The race model was therefore considered violated when the CP of the participant’s RT to the AV stimulus was larger than that predicted by the race model at any quantile within the first 35% of the distribution (represented by the first seven quantiles). It is important to note that failure to violate the race model is not evidence that the two information sources did not interact, but rather it places an upper boundary on RT facilitation that can be accounted for by probability summation.

A “Miller Inequality” value is calculated by subtracting the value predicted by the race model from this CP value, and positive values represent the presence of race model violation. To test the reliability of these effects at the group level, for each of the three groups of participants, Miller Inequality values were submitted to a *t*-test (separately for each of the first seven quantiles). In order to directly test between-group differences in race model violations a one-way between groups ANOVA was computed, such that, for each participant the maximum Miller inequality within the first 35% of the distribution was used as the dependent variable.

### Electroencephalography Acquisition

High-density EEG was recorded from 70 scalp electrodes at a digitization rate of 512 Hz using the BioSemi system. The continuous EEG was recorded referenced to a common mode sense (CMS) active electrode and driven right leg (DRL) passive electrode. CMS and DRL, which replace the ground electrode used in conventional systems, form a feedback loop, thus rendering them references. Offline, the EEG was rereferenced to an average of all electrodes and divided into 1000-ms epochs (200-ms prestimulus to 800-ms post-stimulus onset) to asses slow wave activity in the data and perform high-pass filtering of the data without distorting the epoch of interest (−100 to 500 ms). The low-pass filter was set at 45 Hz, and the high-pass filter at 1.6 Hz. This high-pass setting was selected to avoid spurious MSI effects when comparing the sum to the multisensory response. That is, slow anticipatory activity in the pre-stimulus period (reflecting anticipation of the upcoming target), were they present, would be doubly represented in the summed response, and baseline correction would shift this artifactual difference into the post-stimulus period, leading to such spurious effects (Molholm et al., 2002; Teder-Salejarvi et al., 2002). The anticipatory activity of the kind likely in this scenario is observed at a low frequency (>0.5 Hz) while the dynamics of the event-related potentials (ERPs) of interest are on a much faster time scale. An automatic artifact rejection criterion of ±120 μV from −100 to 500 ms was applied offline to exclude epochs with excessive electromuscular activity. Trials that did not meet criteria for inclusion in the behavioral analyses (described above) were also excluded from the ERP analysis. Electrode channels with excessive noise were interpolated on a trial-by-trial basis using the nearest neighbor spline (Perrin et al., 1987, 1989). Channels with a standard deviation of less than 0.5 μV across the block were interpolated on a block-by-block basis. Finally, if there were more than four bad channels in a trial, the trial was rejected (i.e., no more than four channels were interpolated for any given trial). To compute ERPs, epochs were sorted according to stimulus condition and averaged for each participant. For each participant, the “sum” condition was created by summing the ERPs from the auditory-alone and the visual-alone conditions. Baseline was defined as the epoch from negative −50 to 10 ms relative to stimulus onset, for consistency with our previous work using this paradigm (Molholm et al., 2002; Brandwein et al., 2011, 2013).

### Electrophysiological Analysis

The statistical approach was grounded in prior work from our laboratory using this same paradigm in developmental and clinical cohorts (Brandwein et al., 2013). The amplitude and corresponding topographical foci of the major auditory and visual sensory components served to constrain statistical analyses of group differences in auditory and visual sensory processing, whereas MSI was tested for windows and regions guided by findings in our prior developmental datasets (Brandwein et al., 2011, 2013).

The peak latency of a given unisensory component (as observed in the grand mean data) for each of the participant groups defined the window around which a component’s amplitude was measured in the individual subject data. Amplitude values from each unisensory condition for each time-window of analysis were entered into separate ANOVAs with diagnostic group (TD, ASD, and SPD) as a between participant factor, and, in certain cases, region of interest as a within participant factor. When appropriate, Greenhouse–Geisser corrections were used to report ANOVA results.

Multisensory integration was assessed by comparing the response to the audiovisual condition (AV) to the sum of the responses to the respective unisensory conditions (SUM). Because electric fields sum linearly, divergence between the sum and multisensory responses indicates that the inputs were processed differently when presented together compared to when presented alone. From this, it is inferred that the inputs interacted during neural processing. This assumption is only valid during sensory processing stages. Once neural processes common to each of the unisensory responses begin (such as premotor or motor activity related to making a response), it is no longer valid, since these will be represented twice in the summed response. This represents a common approach to assaying MSI in scalp recorded electrophysiological data (Giard and Peronnet, 1999; Foxe et al., 2000; Teder-Salejarvi et al., 2002; Quinn et al., 2014). Of note, this approach is blind to pure unisensory processing differences since the unisensory responses are, in essence, subtracted out. A mixed-design ANOVA with between participant factor of diagnostic group (TD, ASD, SPD) and within participant factors of condition (AV, SUM) was used to assess MSI in the EEG data.

### *Post hoc* Exploratory Analyses of Sensory Processing Differences and Multisensory Effects

We undertook a secondary exploratory statistical approach to more fully characterize the data and guide hypothesis formulation for future work. Statistical Cluster Plots (S) were generated to assess group differences in unisensory processing, and to fully characterize multisensory effects for each of the groups. Point-wise, unpaired two tailed *t*-tests between comparator conditions were generated for each time point and electrode. As in previous studies (Molholm et al., 2002; De Sanctis et al., 2009; Butler et al., 2012), Type I errors were minimized by only considering a comparison statistically significant if *p* < 0.05 for 11 consecutive data points across adjacent channels (Guthrie and Buchwald, 1991).

In each ANOVA we included Levene’s test for equality of variances, which tests the null hypothesis that the population variance among the sample groups is equal. For each ANOVA reported, Levene’s test did not indicate a rejection of the null hypothesis that the sample population variances were equal (all *p* > 0.05) except in one case. In this case we applied the non-parametric independent-samples Kruskal–Wallis test, which does not assume equality of variance.

## Results

### Behavior

#### Reaction Time

**FIGURE 1:**
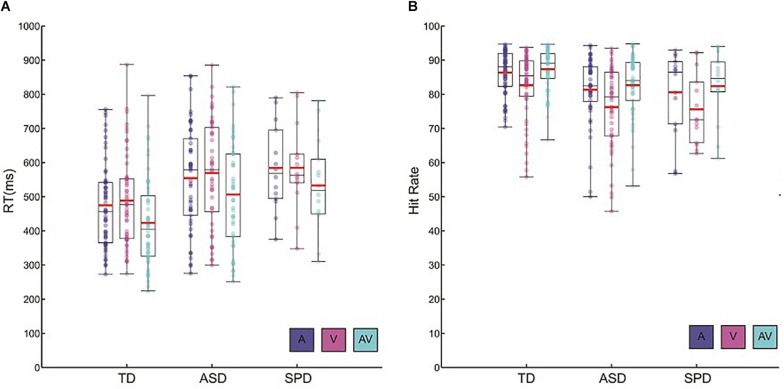
Behavioral data. **(A)** Reaction time data for each of the diagnostic groups and the three stimulus conditions. Circles represent each participant’s mean RT for a given condition. Red horizontal bars indicate the mean across participants within a given group and condition. Black rectangles represents 25th and 75th group percentile bounds, and the horizontal black line within each rectangle is the group median. Whiskers extend to the minimum and maximum data points. **(B)** Hit rate data for each diagnostic group and condition. Plotting conventions are the same as those used for the RT data.

The mean RTs for each of the stimulus modalities suggested a RSE for each of the three groups (Figure 1A). The 3 × 3 mixed model ANOVA with within-participant factor Modality and between-participant factor Diagnostic Group indicated a main effect of Modality (*F*(2,220) = 109.51, *p* < 0.001). Follow-up pairwise comparisons indicated that RT was faster for the AV condition (*M* = 467.81, SD = 145.16) compared to the A (*M* = 519.27, SD = 143.61; *p* < 0.001) and the V (*M* = 530.64, SD = 146.22; *p* < 0.001) conditions. Mean RT was not significantly different among the A and V conditions (*p* = 0.32). Furthermore, the interaction among Modality and Diagnostic Group did not approach significance (*F*(4,220) = 0.42, *p* = 0.75). Together these results point to a similar pattern and magnitude of RSEs among the three diagnostic groups. In addition to the main effect of Modality, the factor Diagnostic Group was also statistically significant (*F*(2,110) = 5.42, *p* = 0.006). Pairwise comparisons indicated that, on the whole, TD participants were faster to respond regardless of stimulus modality relative to both participants in the ASD group (*p* = 0.02) as well as participants in the SPD group (*p* = 0.04). Response times were not significantly different among the ASD and SPD groups (*p* > 0.999). On average, TD participants were 77 ms faster to respond compared to the ASD participants, and 105 ms faster than the SPD participants.

#### Hit Rate

Hit rate among the groups and across the sensory modalities largely paralleled the patterns found in the RT data (Figure 1B). There was a main effect of Modality (*F*(2,220) = 49.39, *p* < 0.001). Bonferroni corrected follow-up pairwise comparisons indicated that, across the diagnostic groups, the AV condition elicited the highest hit rate (*M* = 84.94, SD = 8.35), significantly higher than both the A condition (*M* = 83.64, SD = 9.16; *p* < 0.001) as well as the V condition (*M* = 79.32, SD = 11.21; *p* < 0.001). On the whole, participants had significantly higher hit rates within the A condition relative to the V condition (*p* < 0.001). As in the analysis of the RT data, there was no indication of a significant interaction among Diagnostic Group and Stimulus Modality (*F*(4,220) = 0.72, *p* = 0.58). Hit rate differed among the groups (*F*(2,110) = 5.35, *p* = 0.006), such that TD participants (*M* = 85.44, SD = 7.03) had higher hit rates than ASD participants (*M* = 80.22, SD = 10.05; *p* = 0.004) and SPD participants (*M* = 79.53, SD = 10.31; *p* = 0.026). Overall, all groups were faster and more accurate when redundant audiovisual stimuli were presented relative to the presentation of auditory or visual stimuli alone. Across all of the stimulus conditions, TD participants tended to respond faster and demonstrated higher hit rates than ASD and SPD participants.

#### Testing the Race Model

Race model violations were considered within the first seven quantiles (35%) of the reaction time distribution, since this is within this timeframe that AV interactions are expected prior to fulfillment of a decision criterion within one of the modalities alone (Miller, 1982; Ulrich et al., 2007; Brandwein et al., 2013). Individual subject analysis of the reaction time distributions for each group showed that 42 of the 54 (78%) typically developing children, 8 of the 14 (57%) children with SPD, and 28 of the 45 (62%) children with ASD violated the race model in at least one of the first seven quantiles.

**FIGURE 2:**
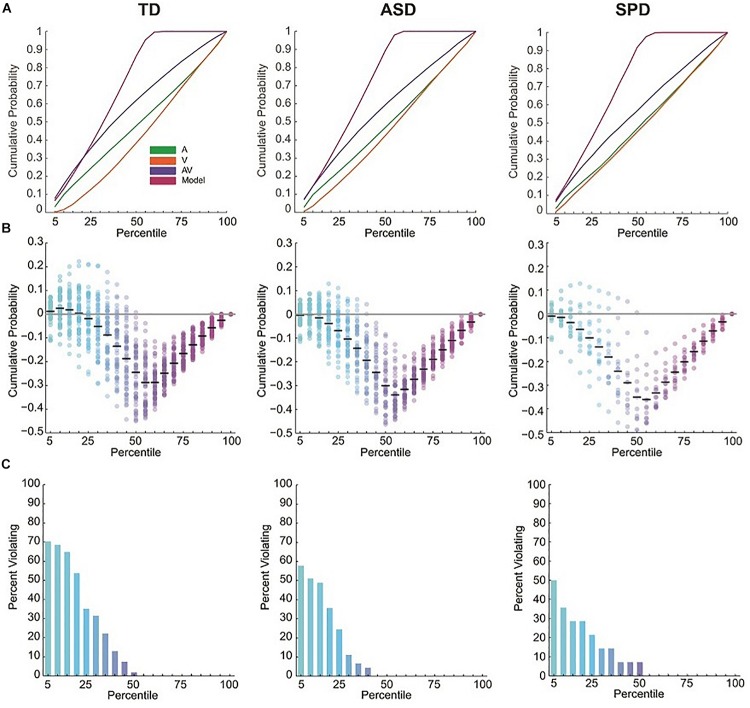
RT cumulative probability distributions and miller inequalities. **(A)** RT cumulative probability for each of the three stimulus conditions and the Race Model. **(B)** Miller inequalities. Semi-opaque circles represent individual participants. Black horizontal bars are the mean across participants at each percentile. **(C)** Percent of participants violating the Race Model at each percentile.

For a given quantile, no reliable race model violations were found in the SPD or ASD groups (Figure 2 and see Supplementary Table S1). This was the case even before Bonferroni correction for multiple tests. In contrast, the race model was reliably violated across participants in the 10th percentile (the second quantile) in the TD group (and in the two surrounding quantiles before Bonferroni correction). The one-way between groups ANOVA comparing the maximum race model violations among the three groups indicated a difference among the diagnostic groups (*F*(2,110) = 5.13, *p* = 0.007). Pairwise comparisons indicated that while ASD (*M* = 0.012, SD = 0.049) and SPD (*M* = 0.006, SD = 0.053) groups did not significantly differ in race model violation (*p* = 0.751), the TD (*M* = 0.044, SD = 0.063) participants demonstrated significantly greater race model violations when compared to both the ASD (*p* = 0.005) and SPD (*p* = 0.027) groups. Thus race-model violation was greatest for the TD group, and did not differ between the ASD and SPD groups.

### Electrophysiology

#### Auditory Alone Responses

**FIGURE 3:**
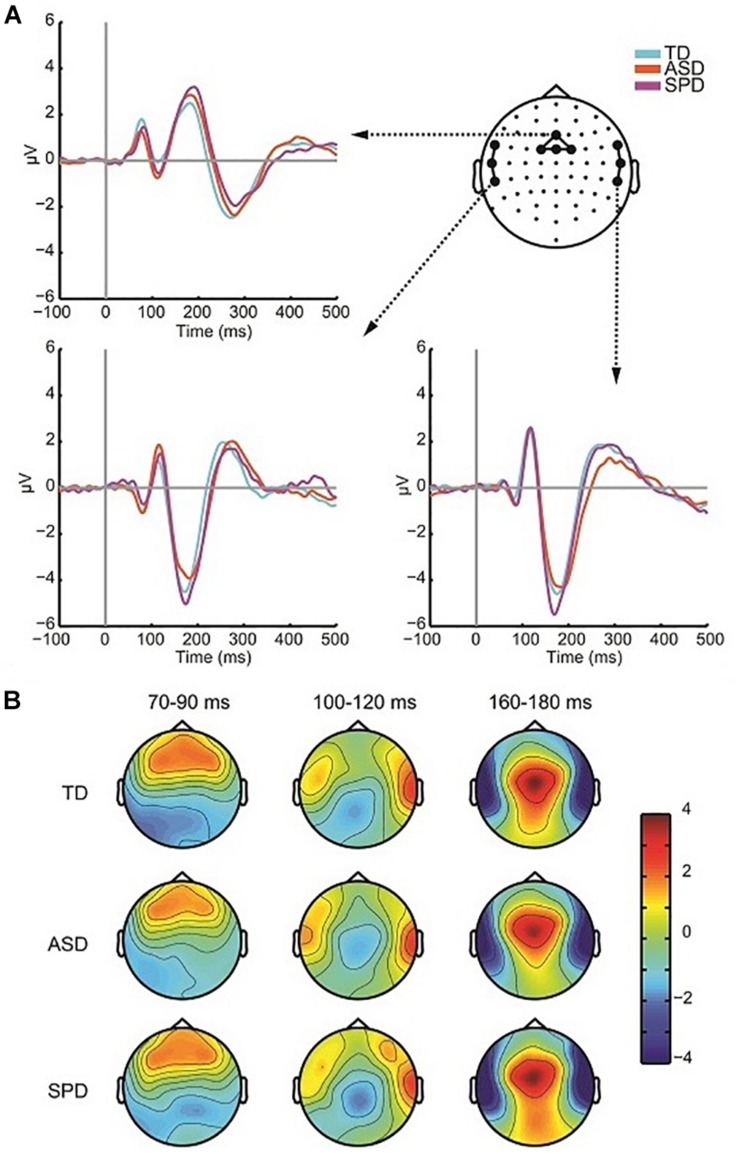
Auditory responses. **(A)** ERP waveforms in response to the auditory alone condition over fronto-central region as well as left and right temporal regions for each of the three diagnostic groups. **(B)** Voltage topographies during temporal windows of interest for each diagnostic group.

The grand mean ERP across all diagnostic groups in response to the Auditory Alone condition showed a typical (for this large age range) auditory P1-N1-P2 complex with foci centered over Fronto-Central, Central, and Temporal scalp regions (Figure 3A). The first apparent activity above baseline was a positivity peaking at ∼80 ms (P1) over fronto-central sites, followed by a negativity peaking at ∼110 ms (N1-Central) over central sites, a negativity peaking at ∼175 ms over left and right temporal sites (N1-Temporal), and lastly a broader positivity peaking at ∼180 ms (P2) over Central sites. The response topographies for each of these timeframes were highly similar across the groups (Figure 3B). Separate ANOVAs were performed for each of these components to assess differences in the AEP among the three diagnostic groups. As can be seen in the analyses reported below, despite the appearance of small differences in the amplitude of the AEP, the planned tests did not reveal any reliable group differences.

To assess the presence of differences in P1 amplitude among the diagnostic groups, a one-way ANOVA was performed. For each participant the average amplitude was computed within the window spanning 60 to 95 ms among a cluster of four fronto-central electrodes (AFZ, FZ, F1, F2). The ANOVA indicated no differences among the three groups (*F*(2,110) = 0.410, *p* = 0.665). The Frontocentral N1, computed as the average spanning the window 92–132 ms over a cluster of four electrodes (Cz, FC1, FCz, FC2), did not differ in amplitude significantly across the three groups (*F*(2,110) = 1.979, *p* = 0.143). The ANOVA on the temporal N1 included data spanning 165–185 ms, and additionally had the factor Hemisphere (Left Temporal, Right Temporal) as the temporal N1 is distributed bilaterally. Three electrodes from each hemisphere were used to compute the mean amplitude over the time window (Left: FT7, T7, TP7; Right: FT8, T8, TP8). The null hypothesis of Levene’s test was rejected for the analysis of the auditory temporal N1 in the 165–185 ms time period due to a significant violation of the equality of variances assumption for the N1 over left hemisphere sensors (*F*(2,110) = 3.605, *p* = 0.030). Running the non-parametric independent-samples Kruskal–Wallis test, which does not assume equality of variance, in a pairwise fashion for left and right hemispheres indicated no significant difference in the auditory N1 among the groups (right: χ^2^(2) = 1.766, *p* = 0.414; left: χ^2^(2) = 2.695, *p* = 0.260). The P2 comprised a positivity over fronto-central electrodes, peaking at ∼180 ms. A window of 160–200 ms and four electrode locations (FCz, FC1, FC2, Cz) were employed to compute mean amplitude. P2 amplitude did not significantly differ across the diagnostic groups (*F*(2,110) = 0.329, *p* = 0.721). Table 3 provides mean amplitude values for the different groups and measures.

**TABLE 3:** Mean (and standard deviation in parentheses) amplitude for each of the Auditory Alone time windows analyzed. All units are in microvolts.

Group	P1	Frontocentral N1	Temporal N1		P2
			Left	Right	
TD	1.71 (1.29)	0.25 (1.84)	−4.38 (2.73)	−4.52 (3.00)	2.96 (1.79)
ASD	1.52 (1.19)	−0.41 (1.47)	−3.68 (2.62)	−4.04 (2.02)	3.00 (1.62)
SPD	1.45 (1.22)	−0.21 (1.57)	−4.88 (3.34)	−5.22 (3.44)	3.40 (2.47)

#### Visual Alone Responses

**FIGURE 4:**
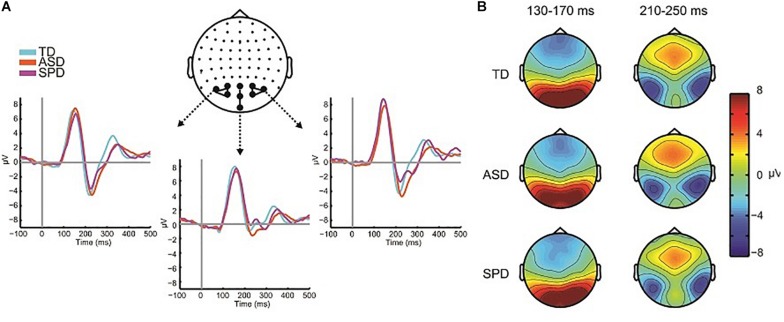
Visual evoked potentials. **(A)** ERP waveforms in response to the visual alone condition over central and lateral occipital scalp regions for each of the three diagnostic groups. **(B)** Voltage topographies during temporal windows of interest for each diagnostic group.

The grand mean of the ERP to the visual alone stimulus showed the expected P1-N1 complex, and was of similar morphology across all three groups (Figure 4A). As can be seen in the scalp topographic maps (Figure 4B), activity was dominant over bilateral posterior scalp sites. A robust P1 peaked at ∼150 ms over left, right and central occipital and parieto-occipital regions, and the visual N1 peaked at ∼220 ms over left and right parieto-occipital regions. To test for differences in the visual responses among the diagnostic groups we followed the same procedure as for the auditory alone condition. The peak of a component was identified both spatially and temporally in the grand mean data and then amplitude values were averaged over the time window centered on the peak activation. As with the analysis of the auditory response, our *a priori* analyses did not reveal group level differences in the VEP response. The analyses and results are described in the following.

For analysis of the P1, two clusters of electrodes were chosen, a left parieto-occipital group (PO3, PO7, O1), and a corresponding right parieto-occipital group (PO4, PO8, O2). The average activity in these regions was then computed for the time window 130–170 ms. The mixed model ANOVA with participant factor Region (Left Parietal-Occipital, Right Parietal-Occipital) and between participant factor Diagnosis showed a significant main effect of Region (*F*(2,220) = 13.187, *p* < 0.001). The main effect of region reflects laterality differences in the amplitude of the P1 such that amplitude is generally greater over right hemisphere electrodes. The main effect of Diagnostic Group was not statistically significant (*F*(2,110) = 0.061, *p* = 0.941), nor was the interaction of Group x Region (*F*(2,110) = 2.162, *p* = 0.120). The next major deflection was seen in the N1 response, with negative foci maxima over left and right occipital regions, peaking at ∼220 ms. Corresponding average amplitude was computed over the time window 190–250 ms for left (P5, P7, P9, PO7) and right (P6, P8, P10, PO8) sensor groups. A mixed model ANOVA with factors Region (Left, Right) and Diagnostic Group showed a main effect of Region (*F*(1,110) = 8.086, *p* = 0.005), reflecting a greater N1 negativity over right occipital scalp compared to left. The main effect of Diagnostic Group did not reach statistical significance (*F*(2,110) = 0.925, *p* = 0.400), nor did the interaction of Group x Region (*F*(2,110) = 0.532, *p* = 0.589). Mean amplitude values for the different groups for the visual P1 and N1 are in Table 4.

**TABLE 4:** Mean (and standard deviation in parentheses) amplitude for each of the Visual Alone time windows analyzed. All units are in microvolts.

Group	P1		N1	
	Left	Right	Left	Right
TD	6.66 (3.74)	7.74 (3.92)	−4.41 (3.44)	−5.01 (3.50)
ASD	6.86 (2.98)	7.15 (3.14)	−3.36 (3.13)	−4.55 (3.84)
SPD	6.05 (2.59)	7.81 (3.81)	−3.36 (2.21)	−4.30 (3.18)

#### Electrophysiological Indices of MSI

**FIGURE 5:**
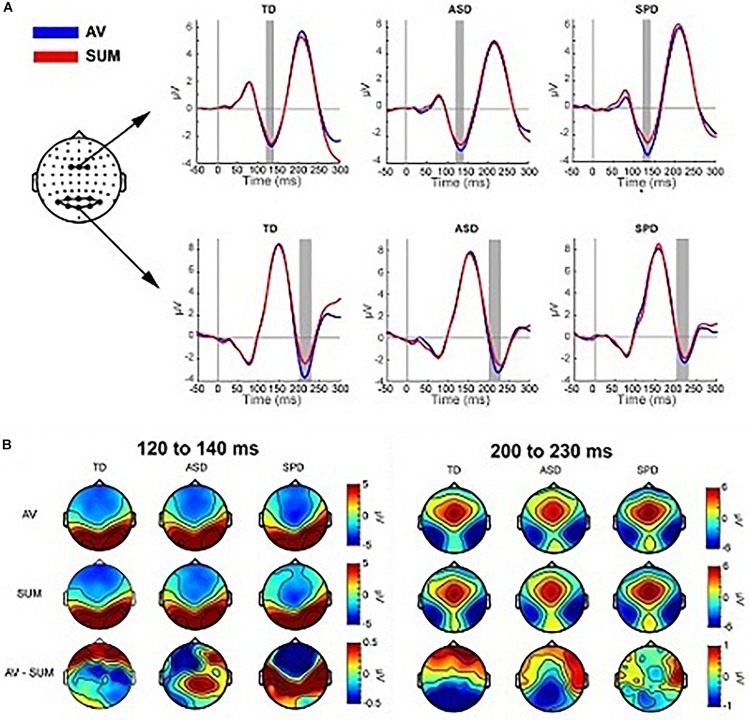
Multisensory effects. **(A)** Grand average waveforms for the AV and SUM conditions averaged over the two clusters of electrodes used in the planned comparisons. The gray rectangles indicate the time range used to compute the average amplitude in the analyses. **(B)** Topographies averaged over the two time windows depicted in panel **(A)**. The top row shows the topographic distribution in the AV condition, the middle row shows the SUM condition and the bottom row depicts their difference (AV *minus* SUM).

Previous studies (Brandwein et al., 2011, 2013) reveal multisensory interactions (i.e., AV ≠A + V) over fronto-central scalp around 120 ms and over left and right parieto-occipital areas around 200 ms (see Figure 5). For the current data windows of analysis were set from 120 to 140 and 200 to 230 ms, over fronto-central and parieto-occipital scalp regions, respectively, such that they centered on the peak amplitudes of the evoked responses.

#### Fronto-Central MSI 120–140 ms

The mixed effects ANOVA in the time window of 120–140 ms over three fronto-central electrodes (FC1, FCz, FC2) indicated a main effect of Condition (*F*(1,110) = 11.164, *p* = 0.001), due to a more negative going response in the AV condition (*M* = −2.86, SD = 2.06) relative to the SUM condition (*M* = −2.51, SD = 2.30). The main effect of Diagnostic Group was not significant (*F*(2,110) = 0.149, *p* = 0.862), nor was the interaction of Condition x Diagnostic Group (*F*(2,110) = 1.479, *p* = 0.232).

#### Parieto-Occipital MSI 200–230 ms

Eight parieto-occipital electrodes were used in the analysis of the posterior negativity (PO7, PO3, POz, PO4, PO8, O1, Oz, O2). The mixed effects ANOVA indicated a main effect of Condition (*F*(1,110) = 13.957, *p* < 0.001) such that the AV condition was more negative (*M* = −2.63, SD = 4.28) than the SUM condition (*M* = −1.73, SD = 4.16). The main effect of Group was not statistically significant (*F*(2,110) = 0.480, *p* = 0.620), nor was the interaction of Condition x Group (*F*(2,110) = 1.236, *p* = 0.295).

### Exploratory Analyses: Statistical Cluster Plots

#### Auditory Alone

**FIGURE 6:**
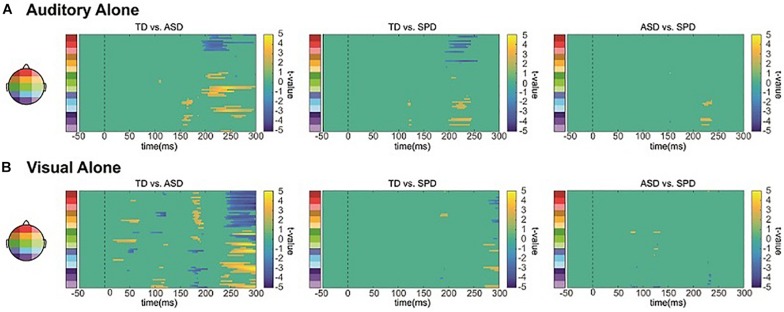
Unisensory SCPs. **(A)** Statistical cluster plots comparing the response to the auditory alone condition between the three diagnostic groups. **(B)** Statistical cluster plots comparing the response to the visual alone condition between the three diagnostic groups.

The between group SCPs comparing the unisensory auditory responses are depicted in Figure 6A. Group differences over right lateral temporal regions in the timeframe of the temporal-N1 (∼170 ms) were apparent between the TD and ASD groups (see also Figure 3A). Additional differences between the TD group and each of the ASD and SPD groups were apparent starting at ∼200 ms, with a hint of a difference between ASD and SPD at ∼225 ms.

#### Visual Alone

The between group SCPs comparing the unisensory visual responses are depicted in Figure 6B. Differences in the visual evoked response are most apparent between the TD and ASD group, at ∼50, 100, and 170 ms, whereas there is little evidence for statistically significant differences between the SPD group and either the TD or the ASD group.

##### Summary of group unisensory processing differences

While the auditory and visual responses were highly similar across the three groups of participants, they also exhibited small amplitude differences. Our planned tests did not reveal any significant differences, yet in applying the less conservative statistical SCP method, we find evidence that both auditory and visual processing differ in ASD compared to a healthy control group (as in, e.g., Brandwein et al., 2013). In contrast, for the SPD group only auditory processing differed significantly, and in this case most compellingly from the TD group. Of course, these data must be considered with caution because they are based on *post hoc* tests. Nevertheless, the large sample sizes for the ASD and TD groups lend confidence to the finding that sensory processing was atypical in the ASD group. In contrast, this analysis only revealed later differences for auditory processing between the SPD and TD groups. Of course it should be noted that this more delimited difference may be due to the smaller sample size in the SPD group, which would decrease sensitivity to detecting real but small effects.

#### Within Group AV Versus SUM Comparisons

**FIGURE 7:**
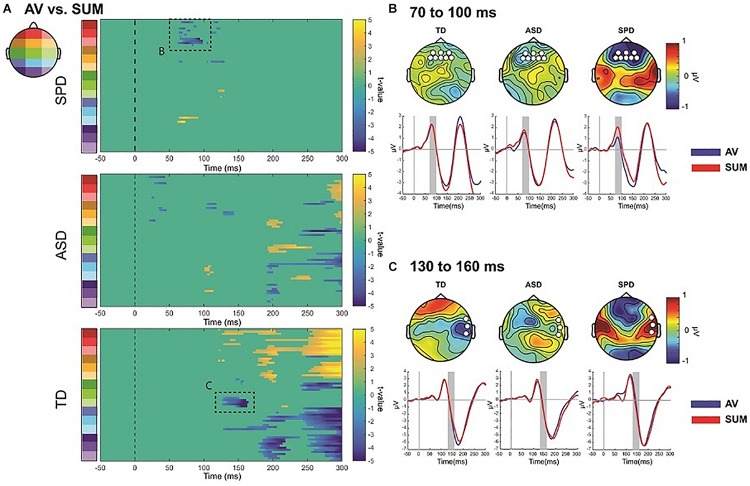
AV versus Sum SCPs, and illustration of follow-up *post hoc* effects. **(A)** Statistical cluster plots comparing AV to SUM conditions for each of the three diagnostic groups. Dashed boxes represent effects that were followed up in *post hoc* ANOVAs across the groups. The letters next to the dashed boxes correspond to the right side panels **(B,C)**. **(B)** Illustration of the 70–90 ms period of interest. Topographies are difference topographies (AV *minus* SUM). The waveforms are the AV and SUM waveforms averaged over the electrodes indicated by the white circles on the corresponding topographies. The time-period of interest is indicated by the gray shaded rectangles. The red trace represents the sum response and the blue trace the multisensory response. **(C)** Illustration of the 130–160 ms effect. Conventions are the same as those in panel **(B)**.

The SCPs comparing the AV condition to the SUM condition revealed differing patterns across the three diagnostic groups (see Figure 7A). We focus here on two spatiotemporal clusters that appear to differ across the groups based on the respective durations of the effect as well as the number of electrodes involved. There were also apparent differences at about 200 ms, with the TD group showing the most robust MSI effects, the ASD group showing weaker but still present MSI effects, and a lack of MSI effects in the SPD group. A planned analysis revealed significant MSI effects in this timeframe, which did not interact with group, and thus this was not followed-up.

The SPD group showed a significant difference between the multisensory and sum conditions from ∼70 to 100 ms over frontal as well as a small region over parieto-occipital scalp (Figure 7). This effect was not apparent in the SCPs of the TD or ASD groups. A *post hoc* ANOVA was run to evaluate this apparent group difference using the average amplitude in the timeframe of 70–100 ms and over a group of frontal and anterior frontal electrodes (AF3, AFZ, AF4, F3, F1, FZ, F2, F4). A mixed model ANOVA with within group factor Condition and between group factor Diagnosis indicated a main effect of Condition (*F*(1,110) = 7.155, *p* = 0.009) as well as an interaction of Group by Condition that approached significance (*F*(2,110) = 2.969, *p* = 0.055), with the SPD group significantly differing from both the TD group (*t*(66) = −2.523, *p* = 0.014) and the ASD group (*t*(57) = −2.018, *p* = 0.048). In contrast, the MSI effect in this timeframe and region was not significantly different between the TD and ASD groups (*t*(97) = 0.568, *p* = 0.571). Inspection of Figure 7B indicates that the AV and SUM waveforms are largely overlapping in this time-period over frontal and anterior frontal regions in the TD and ASD groups, whereas in the SPD group the positive going deflection is clearly larger in the SUM compared to the AV condition. Of course these *post hoc* analyses must be considered with caution.

Relative to the other two diagnostic groups, the TD group showed an initial significant difference beginning at ∼128 ms over right temporal and anterior frontal regions (Figure 7A). This spatiotemporal pattern was not present in the SCPs of the ASD or SPD group. A *post hoc* ANOVA using a cluster of right temporal electrodes (FT8, T8, TP8) averaged over 130–160 ms with within participant factor of Condition and between participant factor Diagnostic Group indicated a significant Condition by Diagnostic Group interaction (*F*(2,110) = 4.965, *p* = 0.003). Follow-up comparisons were performed on the difference between the AV and SUM conditions using between group *t*-tests. This revealed a significant difference in MSI in the TD versus the ASD group (*t*(97) = −2.151, *p* = 0.034) as well as versus the SPD group (*t*(66) = −3.262, *p* = 0.002). The comparison between the ASD and SPD groups did not surpass statistical significance (*t*(57) = −1.802, *p* = 0.077). This pattern of effects was driven by the fact that the TD Group had a more negative going right temporal N1 in the AV condition compared to the SUM condition (AV: *M* = −2.17, SD = 3.32; SUM: *M* = −1.74, SD = 3.29), in the SPD group this pattern was reversed (AV: *M* = −0.57, SD = 3.31; SUM: *M* = −1.39, SD = 2.86). In the ASD group the pattern was also reversed relative to the TD group, but the difference between conditions was relatively small (AV: *M* = −1.42, SD = 2.41; SUM: *M* = −1.56, SD = 2.46).

## Discussion

The neurobiological basis of SPD, and of pathological sensory reactivity in general is, as yet, not well understood. Prior work, however, implicates posterior neural pathways (including the posterior corpus callosum, left posterior thalamic radiations, left posterior corona radiata, and the posterior aspect of the left superior longitudinal fasciculus) in SPD that are associated with sensory processing and MSI (Owen et al., 2013; Chang et al., 2014). While the functional consequences remain to be thoroughly characterized, impaired communication across the sensory systems and decreased MSI could be one result (Chang et al., 2015). This of course fits well with the SPD phenotype of maladaptive responses to the sensory environment. That is, if the myriad inputs to the sensory systems are not integrated into coherent units, they may be experienced as overwhelming. We therefore tested whether individuals with SPD in fact show behavioral evidence for deficits in MSI, and, using high-density electrophysiological recordings of brain activity, whether impaired MSI was evident at early stages of information processing. Inclusion of an ASD group allowed us to determine if any observed differences were specific to the SPD group, or might instead represent a more general characteristic of the sensory reactivity phenotype.

Behaviorally, the SPD group showed reduced MSI compared to the TD group. This was similar to the reduced MSI observed in the ASD group. At the group level, violation of the race model, our behavioral metric of MSI, was not observed in either the SPD or the ASD groups, whereas it was present in the TD group. Comparing maximum RMV across the groups for the early range of the distribution, RMV was smaller for the SPD and ASD groups compared to the TD group. Thus, children with SPD and with ASD simply do not benefit at an age appropriate level from multisensory inputs.

Based on these behavioral data, we might expect diminished neural indices of MSI in the SPD and ASD groups. However, in the electrophysiological data, MSI was present in all groups, and initial *a priori* planned analyses failed to reveal group differences. MSI has a protracted developmental trajectory (Brandwein et al., 2011; Ross et al., 2011, 2015; Foxe et al., 2015), with relatively dramatic changes observed across the age-span of the participants reported in the current study (i.e., 5–15) in both the underlying neurophysiology and in the behavioral benefits that multisensory inputs provide. Notably, a large-scale ASD study in which we were able to divide the participants into different age groups revealed neural differences in MSI (Brandwein et al., 2013). With a limited sample of 14 SPD participants in the present study and a large age-range, a similar approach was not possible and undoubtedly weakened our sensitivity to MSI effects, and to differences in MSI between groups. *Post hoc* analyses supported group differences from 130 to 160 ms, with greater MSI in the TD than either SPD or ASD groups. Given that this *post hoc* finding is for a modest sample size, at least for the SPD group, this finding clearly requires replication before drawing major conclusions with regard to the neurophysiology of MSI in these clinical groups. That said, this pattern would fit the reduced behavioral MSI effects for the SPD and ASD groups. Our *post hoc* observation of a period of greater MSI processing in the SPD group during the earlier timeframe of 70–100 ms is also intriguing, but again, should be considered with caution.

These data additionally provide a window into the neural processing of auditory and visual stimuli in individuals with SPD. While observations made here may not apply to different types of stimuli (e.g., inputs that might be rated as noxious by an individual with SPD), it is the similarity of the basic sensory response across the three groups that stands out. Across the three participant groups, the auditory and visual sensory evoked responses were highly similar in latency and topography, showing only small differences in amplitude. This is evident in the depiction of the AEP in Figure 3, in which the peak latencies of the responses and the topographies of the major deflections at three time points appear wholly similar. Likewise, as seen in waveforms and topographies of the VEP depicted in Figure 4, the latencies and topographies of the peak amplitudes of the VEPs were highly similar across the groups. Both *a priori* analyses and *post hoc* SCPs supported that the auditory and visual responses of the SPD group did not differ in any substantive manner from those in either the TD or ASD groups. These findings suggest that basic sensory registration and early sensory-perceptual processing is largely typical in SPD for these types of stimuli. Of note, the present study was likely only powered to observe large effect sizes in comparisons made between TD and SPD cohorts, whereas considerably smaller effects could be detected in the ASD v. TD comparisons due to the substantially larger cohorts in those groups. Nevertheless, consistent with our findings, a recent magnetoencephalographic study found that early somatosensory and auditory evoked responses were highly similar across SPD and TD groups (Demopoulos et al., 2017). To test for subtle sensory processing differences in SPD, appropriately powered studies in which a greater density of high quality data is collected will be critical. That said, our data and others’ are consistent with early sensory and multisensory processing being largely intact in SPD. Thus it may be later cognitive processes, and/or modulation of the ongoing sensory input, that lead to the sensory reactivity characteristic of SPD, and that yield the behavioral differences observed here, as well as in a companion study in which we find that integration of audio-visual speech is also greatly reduced in SPD (see Foxe et al., current issue).

### Study Considerations

In considering these data, certain study design features and limitations are of note. The SPD participants were selected for being over-responders. Thus these data come from a subtype of individuals considered to have pathological responses to the sensory environment. We chose to focus on a group where sensory reactivity was a primary complaint. Many complex neurodevelopmental disorders have overlapping symptomatology, including sensory reactivity, and likely overlapping genetic liability. As such, future work may benefit from considering sensory reactivity using a transdiagnostic approach. The age-range of the study participants is large, whereas we did not have an adequate sample size to account for potential developmentally specific differences in SPD. This large age range introduces variability due to developmental effects on the brain and behavioral responses (e.g., Brandwein et al., 2011), which adds variance to the signal of interest.

## Conclusion

Together, the present findings and those in Foxe and colleagues (current issue), have clear functional implications: the inability to fully benefit from multisensory cues to optimize performance results in lower fidelity processing of the environment for the individual with SPD. In contrast, in their entirety, the current electrophysiological data suggest that early sensory processing and integration is largely intact in SPD. Further studies will be needed to identify the neural sources underlying behavioral findings of impaired MSI in SPD. For example, examination of later top-down modulatory process, in a design using stimuli to which the participants are under- or over-reactive, may be a particularly fruitful direction for understanding brain processes underlying pathological sensory reactivity.

## Data Availability Statement

The dataset supporting the conclusions of this article will be made available on request to the authors.

## Ethics Statement

This study was approved by the Institutional Review Board of the Albert Einstein College of Medicine (Protocol Reference Number #2011-210). Written informed consent was obtained from parents or legal guardians, where possible assent from the patient was also ascertained, and all aspects of the research conformed to the tenets of the Declaration of Helsinki.

## Author Contributions

SM and JF designed and implemented the study. The technical team at the CNL collected the bulk of the data. ER recruited and phenotyped the SPD patients. JB performed or supervised the clinical and cognitive testing of the majority of participants. JM performed the main data analyses and produced the data illustrations. SM, JF, and JM discussed and conducted the statistical analyses. SM wrote the first draft of the manuscript and received extensive editorial input on subsequent drafts from all of the co-authors. All authors have evaluated the final version of the manuscript, had full and unfettered access to the datasets used to generate this report, and read and approved the final version of the manuscript.

## Conflict of Interest

The authors declare that the research was conducted in the absence of any commercial or financial relationships that could be construed as a potential conflict of interest.

## Footnotes

1 The SPS assesses seven domains of sensory processing for three different types of abnormality, but for the purposes of this study, only SOR in three chosen domains factored into classification.

## References

[B1] Ayres A. J. (1979). *Sensory Integration and the Child.* Los Angeles, CA: Western Psychological Services.

[B2] Ben-Sasson A. Hen L. Fluss R. Cermak S. A. Engel-Yeger B. Gal E. (2009). A meta-analysis of sensory modulation symptoms in individuals with autism spectrum disorders. *J. Autism. Dev. Disord.* 39 1–11. 10.1007/s10803-008-0593-3 18512135

[B3] Brandwein A. B. Foxe J. J. Butler J. S. Frey H. P. Bates J. C. Shulman L. H. (2015). Neurophysiological indices of atypical auditory processing and multisensory integration are associated with symptom severity in autism. *J. Autism. Dev. Disord.* 45 230–244. 10.1007/s10803-014-2212-9 25245785 PMC4289100

[B4] Brandwein A. B. Foxe J. J. Butler J. S. Russo N. N. Altschuler T. S. Gomes H. (2013). The development of multisensory integration in high-functioning autism: high-density electrical mapping and psychophysical measures reveal impairments in the processing of audiovisual inputs. *Cereb. Cortex* 23 1329–1341. 10.1093/cercor/bhs109 22628458 PMC3643715

[B5] Brandwein A. B. Foxe J. J. Russo N. N. Altschuler T. S. Gomes H. Molholm S. (2011). The development of audiovisual multisensory integration across childhood and early adolescence: a high-density electrical mapping study. *Cereb. Cortex* 21 1042–1055. 10.1093/cercor/bhq170 20847153 PMC3077428

[B6] Butler J. S. Foxe J. J. Fiebelkorn I. C. Mercier M. R. Molholm S. (2012). Multisensory representation of frequency across audition and touch: high density electrical mapping reveals early sensory-perceptual coupling. *J. Neurosci.* 32 15338–15344. 10.1523/JNEUROSCI.1796-12.2012 23115172 PMC3664421

[B7] Chang Y. S. Gratiot M. Owen J. P. Brandes-Aitken A. Desai S. S. Hill S. S. (2015). White matter microstructure is associated with auditory and tactile processing in children with and without sensory processing disorder. *Front. Neuroanat.* 9:169. 10.3389/fnana.2015.00169 26858611 PMC4726807

[B8] Chang Y. S. Owen J. P. Desai S. S. Hill S. S. Arnett A. B. Harris J. (2014). Autism and sensory processing disorders: shared white matter disruption in sensory pathways but divergent connectivity in social-emotional pathways. *PLoS One* 9:e103038. 10.1371/journal.pone.0103038 25075609 PMC4116166

[B9] Davies P. L. Chang W. P. Gavin W. J. (2009). Maturation of sensory gating performance in children with and without sensory processing disorders. *Int. J. Psychophysiol.* 72 187–197. 10.1016/j.ijpsycho.2008.12.007 19146890 PMC2695879

[B10] Davies P. L. Chang W. P. Gavin W. J. (2010). Middle and late latency ERP components discriminate between adults, typical children, and children with sensory processing disorders. *Front. Integr. Neurosci.* 4:16. 10.3389/fnint.2010.00016 20577583 PMC2889678

[B11] De Sanctis P. Molholm S. Shpaner M. Ritter W. Foxe J. J. (2009). Right hemispheric contributions to fine auditory temporal discriminations: high-density electrical mapping of the duration mismatch negativity (MMN). *Front. Integr. Neurosci.* 3:5. 10.3389/neuro.07.005.2009 19430594 PMC2679157

[B12] Demopoulos C. Yu N. Tripp J. Mota N. Brandes-Aitken A. N. Desai S. S. (2017). Magnetoencephalographic imaging of auditory and somatosensory cortical responses in children with autism and sensory processing dysfunction. *Front. Hum. Neurosci.* 11:259. 10.3389/fnhum.2017.00259 28603492 PMC5445128

[B13] Dunn W. (1999). *Sensory Profile: User’s Manual.* San Antonio, TX: The Psychological Corporation.

[B14] Fiebelkorn I. C. Foxe J. J. Butler J. S. Molholm S. (2011). Auditory facilitation of visual-target detection persists regardless of retinal eccentricity and despite wide audiovisual misalignments. *Exp. Brain Res.* 213 167–174. 10.1007/s00221-011-2670-7 21479656

[B15] Fiebelkorn I. C. Snyder A. C. Mercier M. R. Butler J. S. Molholm S. Foxe J. J. (2013). Cortical cross-frequency coupling predicts perceptual outcomes. *NeuroImage* 69 126–137. 10.1016/j.neuroimage.2012.11.021 23186917 PMC3872821

[B16] Foss-Feig J. H. Heacock J. L. Cascio C. J. (2012). Tactile responsiveness patterns and their association with core features in autism spectrum disorders. *Res. Autism. Spectr. Disord.* 6 337–344. 10.1016/j.rasd.2011.06.007 22059092 PMC3207504

[B17] Foxe J. J. Molholm S. (2009). Ten years at the multisensory forum: musings on the evolution of a field. *Brain Topogr.* 21 149–154. 10.1007/s10548-009-0102-9 19452270

[B18] Foxe J. J. Molholm S. Del Bene V. A. Frey H. P. Russo N. N. Blanco D. (2015). Severe multisensory speech integration deficits in high-functioning school-aged children with autism spectrum disorder (ASD) and their resolution during early adolescence. *Cereb. Cortex* 25 298–312. 10.1093/cercor/bht213 23985136 PMC4303800

[B19] Foxe J. J. Morocz I. A. Murray M. M. Higgins B. A. Javitt D. C. Schroeder C. E. (2000). Multisensory auditory-somatosensory interactions in early cortical processing revealed by high-density electrical mapping. *Brain Res. Cogn. Brain Res.* 10 77–83. 10.1016/s0926-6410(00)00024-0 10978694

[B20] Gavin W. J. Dotseth A. Roush K. K. Smith C. A. Spain H. D. Davies P. L. (2011). Electroencephalography in children with and without sensory processing disorders during auditory perception. *Am. J. Occup. Ther.* 65 370–377. 10.5014/ajot.2011.002055 21834451

[B21] Giard M. H. Peronnet F. (1999). Auditory-visual integration during multimodal object recognition in humans: a behavioral and electrophysiological study. *J. Cogn. Neurosci.* 11 473–490. 10.1162/089892999563544 10511637

[B22] Guthrie D. Buchwald J. S. (1991). Significance testing of difference potentials. *Psychophysiology* 28 240–244. 10.1111/j.1469-8986.1991.tb00417.x 1946890

[B23] Mercier M. R. Molholm S. Fiebelkorn I. C. Butler J. S. Schwartz T. H. Foxe J. J. (2015). Neuro-oscillatory phase alignment drives speeded multisensory response times: an electro-corticographic investigation. *J. Neurosci.* 35 8546–8557. 10.1523/JNEUROSCI.4527-14.2015 26041921 PMC6605331

[B24] Miller J. (1982). Divided attention: evidence for coactivation with redundant signals. *Cogn. Psychol.* 14 247–279. 10.1016/0010-0285(82)90010-x 7083803

[B25] Miller L. J. Schoen S. A. James K. Schaaf R. C. (2007). Lessons learned: a pilot study on occupational therapy effectiveness for children with sensory modulation disorder. *Am. J. Occup.* 61 161–169. 10.5014/ajot.61.2.161 17436838

[B26] Molholm S. Ritter W. Javitt D. C. Foxe J. J. (2004). Multisensory visual-auditory object recognition in humans: a high-density electrical mapping study. *Cereb. Cortex* 14 452–465. 10.1093/cercor/bhh007 15028649

[B27] Molholm S. Ritter W. Murray M. M. Javitt D. C. Schroeder C. E. Foxe J. J. (2002). Multisensory auditory-visual interactions during early sensory processing in humans: a high-density electrical mapping study. *Brain Res. Cogn. Brain Res.* 14 115–128. 10.1016/s0926-6410(02)00066-6 12063135

[B28] Owen J. P. Marco E. J. Desai S. Fourie E. Harris J. Hill S. S. (2013). Abnormal white matter microstructure in children with sensory processing disorders. *NeuroImage. Clin.* 2 844–853. 10.1016/j.nicl.2013.06.009 24179836 PMC3778265

[B29] Perrin F. Pernier J. Bertrand O. Echallier J. F. (1989). Spherical splines for scalp potential and current density mapping. *Electroencephalogr. Clin. Neurophysiol.* 72 184–187. 10.1016/0013-4694(89)90180-6 2464490

[B30] Perrin F. Pernier J. Bertrand O. Giard M. H. Echallier J. F. (1987). Mapping of scalp potentials by surface spline interpolation. *Electroencephalogr. Clin. Neurophysiol.* 66 75–81. 10.1016/0013-4694(87)90141-6 2431869

[B31] Quinn B. T. Carlson C. Doyle W. Cash S. S. Devinsky O. Spence C. (2014). Intracranial cortical responses during visual-tactile integration in humans. *J. Neurosci.* 34 171–181. 10.1523/JNEUROSCI.0532-13.2014 24381279 PMC3866483

[B32] Reynolds S. Lane S. J. (2009). Sensory overresponsivity and anxiety in children with ADHD. *Am. J. Occup. Ther.* 63 433–440. 10.5014/ajot.63.4.433 19708472

[B33] Ross L. A. Del Bene V. A. Molholm S. Frey H. P. Foxe J. J. (2015). Sex differences in multisensory speech processing in both typically developing children and those on the autism spectrum. *Front. Neurosci.* 9:185. 10.3389/fnins.2015.00185 26074757 PMC4445312

[B34] Ross L. A. Molholm S. Blanco D. Gomez-Ramirez M. Saint-Amour D. Foxe J. J. (2011). The development of multisensory speech perception continues into the late childhood years. *Eur. J. Neurosci.* 33 2329–2337. 10.1111/j.1460-9568.2011.07685.x 21615556 PMC3127459

[B35] Schaaf R. C. Benevides T. Mailloux Z. Faller P. Hunt J. van Hooydonk E. (2013). An intervention for sensory difficulties in children with autism: a randomized trial. *J. Autism. Dev. Disord.* 44 1493–1506. 10.1007/s10803-013-1983-8 24214165 PMC4057638

[B36] Schaaf R. C. Schoen S. A. Smith Roley S. Lane S. J. Koomar J. A. May-Benson T. A. (2009). *A Frame of Reference for Sensory Integration.* Philadelphia, PA: Lippincott Williams and Wilkins.

[B37] Tavassoli T. Miller L. J. Schoen S. A. Jo Brout J. Sullivan J. Baron-Cohen S. (2017). Sensory reactivity, empathizing and systemizing in autism spectrum conditions and sensory processing disorder. *Dev. Cogn. Neurosci*. 29 72–77. 10.1016/j.dcn.2017.05.005 28579480 PMC6987900

[B38] Teder-Salejarvi W. A. McDonald J. J. Di Russo F. Hillyard S. A. (2002). An analysis of audio-visual crossmodal integration by means of event-related potential (ERP) recordings. *Brain Res. Cogn. Brain Res.* 14 106–114. 10.1016/s0926-6410(02)00065-4 12063134

[B39] Ulrich R. Miller J. Schroter H. (2007). Testing the race model inequality: an algorithm and computer programs. *Behav. Res. Methods* 39 291–302. 10.3758/bf03193160 17695357

