# Supplementary material for: Multisensory Audiovisual Processing in Children With a Sensory Processing Disorder (I): Behavioral and Electrophysiological Indices Under Speeded Response Conditions
Source: Front Integr Neurosci. 2020 Feb 11;14:4. doi: 10.3389/fnint.2020.00004 (PMC7026671; doi:10.3389/fnint.2020.00004)
Supplement: Supplementary file 1 [file Table_1.docx]

| **TD** | | | | | | | |
| --- | --- | --- | --- | --- | --- | --- | --- |
| Percentile | 5^th^ | **10^th^** | 15^th^ | 20^th^ | 25^th^ | 30^th^ | 35^th^ |
|  | *t*(53) = 2.09,  *p* = 0.02* | ***t*(53) = 3.13,**  ***p* = 0.001*^#^** | *t*(53) = 1.86,  *p* = 0.03* | *t*(53) = 0.30,  *p* = 0.38 | *t*(53) = -1.48,  *p* = 0.93 | *t*(53) = -3.55,  *p >* 0.99 | *t*(53) = -5.76,  *p >* 0.99 |
| **ASD** | | | | | | | |
| Percentile | 5^th^ | 10^th^ | 15^th^ | 20^th^ | 25^th^ | 30^th^ | 35^th^ |
|  | *t*(45) = -1.05,  *p* = 0.85 | *t*(45) = -0.81,  *p* = 0.79 | *t*(45) = -1.89,  *p* = 0.97 | *t*(45) = -3.40,  *p* > 0.99 | *t*(45) = -5.30,  *p* > 0.99 | *t*(45) = -7.56,  *p* > 0.99 | *t*(45) = -9.55,  *p* > 0.99 |
| **SPD** | | | | | | | |
| Percentile | 5^th^ | 10^th^ | 15^th^ | 20^th^ | 25^th^ | 30^th^ | 35^th^ |
|  | *t*(13) = -0.75,  *p* = 0.76 | *t*(13) = -0.94,  *p* = 0.82 | *t*(13) = -1.78,  *p* = 0.95 | *t*(13) = -2.59,  *p* > 0.99 | *t*(13) = -3.38,  *p* > 0.99 | *t*(13) = -4.22,  *p* > 0.99 | *t*(13) = -5.12,  *p* > 0.99 |

**Race model violation t-statistics**

* Significant at an alpha level of *p < 0.05*

*^#^* Significant after Bonferroni correction for multiple comparisons

Caption for Supplementary Table 1: t-test statistics for each quantile for each group.
